# *De novo* design of new chemical entities for SARS-CoV-2 using artificial intelligence

**DOI:** 10.4155/fmc-2020-0262

**Published:** 2021-02-16

**Authors:** Navneet Bung, Sowmya R Krishnan, Gopalakrishnan Bulusu, Arijit Roy

**Affiliations:** ^1^TCS Innovation Labs-Hyderabad (Life Sciences Division), Tata Consultancy Services Limited, Hyderabad 500081, India

**Keywords:** 3CL protease, artificial intelligence, COVID-19, deep learning, protease inhibitors, SARS-CoV-2

## Abstract

**Background:** The novel coronavirus SARS-CoV-2 has severely affected the health and economy of several countries. Multiple studies are in progress to design novel therapeutics against the potential target proteins in SARS-CoV-2, including 3CL protease, an essential protein for virus replication. **Materials & methods:** In this study we employed deep neural network-based generative and predictive models for *de novo* design of small molecules capable of inhibiting the 3CL protease. The generative model was optimized using transfer learning and reinforcement learning to focus around the chemical space corresponding to the protease inhibitors. Multiple physicochemical property filters and virtual screening score were used for the final screening. **Conclusion:** We have identified 33 potential compounds as ideal candidates for further synthesis and testing against SARS-CoV-2.

The novel coronavirus SARS-CoV-2 has been the causative agent of a global pandemic [1] with a high mortality rate. Epidemics involving two other coronaviruses, namely the severe acute respiratory syndrome virus (SARS-CoV) and the Middle East respiratory syndrome virus (MERS-CoV), have occurred in 2003 and 2012, respectively [2,3]. Despite these past epidemics, the unavailability of promising vaccine candidates and potential therapeutics for coronaviruses has impeded global efforts to control the pandemic.

Coronaviruses are of zoonotic origin and belong to the family Coronaviridae, which consists of four genera; SARS-CoV-2 belongs to the Betacoronaviridae genus and has the largest RNA genome, ∼32 kb in size. The genome of SARS-CoV-2 is ∼96% identical to that of the bat coronavirus [4]. The genomes of all coronaviruses encode two types of proteins, namely the structural and nonstructural proteins. The major structural proteins of all coronaviruses include: spike glycoprotein (S), envelope protein (E), membrane protein (M) and nucleocapsid protein (N) [5,6]. Nonstructural proteins contribute to other essential viral processes, including viral genome replication and viral assembly. Among the structural proteins, the spike glycoprotein interacts with the host cell surface receptor ACE2 and enables the entry of viral genome into the host cell [7,8]. The viral genome gets translated in the host cell cytoplasm into two long polyproteins required for viral replication [9]. These polyproteins are cleaved by viral proteases into various structural and nonstructural proteins; this is an essential post-translational modification required for viral maturation. Among the viral proteases identified in coronaviruses, the chymotrypsin-like (3CL) protease (M^pro^ or main protease) is primarily involved in polyprotein cleavage, while the papain-like protease aids the main protease in the process. Due to their significant contribution to viral entry and viral replication, the spike protein, 3CL protease and papain-like protease are promising drug targets in SARS-CoV-2 [6].

The 3CL protease is a homodimeric cysteine protease [10]. The 3D co-ordinates of 3CL protease are available in the Protein Data Bank (PDB: 6LU7; Figure 1) [10]. The structural superposition of the 3CL protease of SARS-CoV and SARS-CoV-2 shows a root mean square deviation of 0.56 Å, indicating that the structure of the protein is highly conserved. The protein is composed of three domains, and the binding site lies in a cleft between domains I and II [10]. The residues Cys145 and His41 form the catalytic dyad at the binding site of the protein (Figure 1). The mechanism of action of the 3CL protease of SARS-CoV-2 can be inferred based on the mechanism proposed earlier for SARS-CoV (Supplementary Figure 1) [11,12]. Due to the highly conserved nature of the active site of 3CL protease among coronaviruses, covalent inhibitors such as N3, originally designed against SARS-CoV and MERS-CoV, have also been shown to inhibit the 3CL protease of SARS-CoV-2, indicating a broad spectrum of activity [10,13]. Apart from N3, two covalent inhibitors (compounds **11a** and **11b**) have shown promising results *in vitro* and *in vivo* [13].

**Figure 1.**
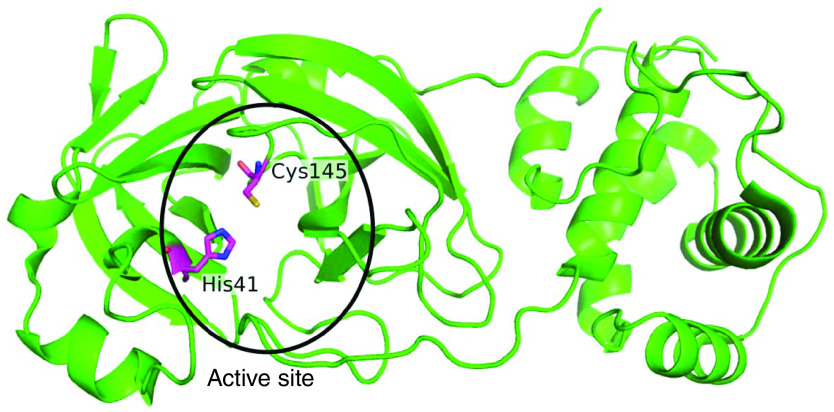
3D structure of 3CL protease from SARS-CoV-2. The active site residues His41 and Cys145 which are crucial for the catalytic process of 3CL protease are shown in magenta sticks.

The majority of the drug discovery efforts against SARS-CoV-2 are focused on repurposing existing antiviral drugs. For example, initial clinical trials against SARS-CoV-2 involved repurposing of existing HIV protease inhibitors such as ASC09, darunavir, indinavir, lopinavir, ritonavir and saquinavir [14]. Although the lopinavir–ritonavir combination therapy (Kaletra) has shown success in initial phases of clinical trials, further studies have shown that the drug shows no benefit for the primary end point beyond standard care in patients with severe COVID-19 [15]. ASC09 is also currently in clinical trials despite the noted lack of specific research associating the drug with COVID-19 [16]. These observations show there is a need for designing better and more potent new chemical entities (NCEs) that can specifically target the 3CL protease of SARS-CoV-2. Fragment-based *de novo* drug design methods [17] with multitasking models for quantitative structure–biological effect relationships have shown some success for antiviral [18] and antimicrobial drug design [19,20]. However, with the recent developments in the field of artificial intelligence (AI), it is possible to mine existing knowledge and use this information to explore the virtually unlimited chemical space and develop novel small molecules with the desired biological and physicochemical properties [21–23]. Notably, AI-based methods have recently been used to develop novel antibacterial molecules [23].

In this study, to design NCEs against the 3CL protease of SARS-CoV-2, knowledge of viral protease inhibitors was used to train the deep neural network-based generative and predictive models. Inhibiting the 3CL protease might hamper viral maturation, thereby reducing SARS-CoV-2 infection in humans.

## Materials & methods

### Data collection

The datasets for training the deep neural network models were collected from the ChEMBL database [24]. A dataset of ∼1.6 million drug-like small molecules was collected for pretraining the generative model. Because there is limited knowledge about small molecules that can inhibit the 3CL protease, a dataset of small molecules which were experimentally verified to inhibit viral proteases was collected from the ChEMBL database. A total of 7665 viral protease inhibitors were collected. Among them, molecules with a pChEMBL score greater than 7.0 were screened at the active site of the 3CL protease of SARS-CoV-2 using AutoDock Vina [25]. In total, 2515 molecules passed the screening test and were considered for retraining the deep neural network models. All the datasets of small molecules were represented using the Simplified Molecular Input Line Entry System (SMILES) format [26], to leverage the effectiveness of recurrent neural networks in handling sequential data.

### Data preprocessing

The SMILES datasets were preprocessed by applying sequential filters to remove stereochemistry, salts and molecules with undesirable atoms or groups [21,27]. SMILES strings >100 symbols in length were removed, as ∼97% of the dataset consists of SMILES strings with <100 symbols [21]. Finally, the dataset was canonicalized to remove redundant small molecules. The RDKit library in Python was used for dataset preprocessing.

All the SMILES strings in the dataset were appended with a start-of-sequence character and an end-of-sequence character at the beginning and end of the sequence, respectively [27]. Finally, the SMILES strings were one-hot encoded using a vocabulary of 39 symbols.

### Learning the language of small molecules using the generative model

**Figure 2.**
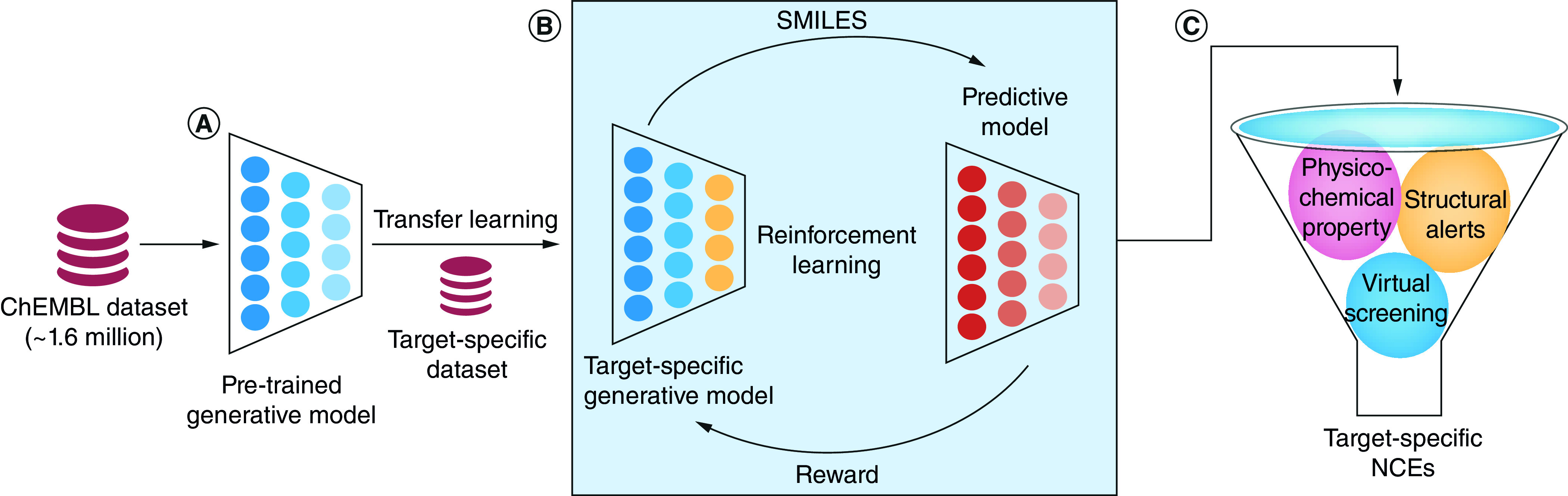
*De novo* drug design pipeline for generating small molecules against a target of interest. **(A)** Pretrained generative model. **(B)** Transfer learning (TL) to learn the features of small molecules specific to the target protein and reinforcement learning (RL) to optimize the property of interest. **(C)** Different physico-chemical property filters, structural alerts and virtual screening score were used for the final screening.

The dataset of ∼1.6 million drug-like small molecules in SMILES format was used for pretraining the generative model (Figure 2A). The deep neural network architecture of the generative model (Supplementary Figure 2A) consists of a single layer of 1024 bidirectional gated recurrent units (GRUs) as the internal memory [28], augmented with a stack acting as the dynamic external memory [29]. Stack augmentation of existing GRU cells [29] improves the capacity of recurrent neural network models in capturing the syntactic and semantic features inherent to the context-free grammar of sequential data [21,30]. Training was performed using mini-batch gradient descent with AMSGrad optimizer [31].

During the inference phase, the start-of-sequence character was given as input to the generative model and the subsequent characters of the SMILES string were sampled one at a time using multinomial sampling. The sampling process was terminated if either the end-of-sequence character was sampled or the length of the SMILES string exceeded a predefined maximum threshold. The chemical validity was checked using the RDKit library for every sampled SMILES string to ensure the synthetic feasibility of the generated small molecule. The model was trained for 500 epochs on a Tesla V100 graphics processing unit (GPU) and the weights from the trained model were used for the downstream tasks in the pipeline (Figure 2). All implementations were done in PyTorch [32].

### Benchmark metrics of the generative model obtained after pretraining

The pretrained generative model was evaluated using the metrics specified in the GuacaMol benchmark for deep generative models [33]. All the metrics were calculated with a dataset of 10,000 molecules sampled from the pretrained generative model. The validity of the generative model was 96.6%, indicating the accuracy of the model in producing chemically feasible SMILES strings. Out of the 10,000 molecules sampled, 99.9% of the small molecules were unique. Only 1.85% of small molecules were identical to the ChEMBL training dataset [24]. The Kullback–Leibler divergence metric for nine physicochemical descriptors compared with the training dataset was found to be 0.991, showing that the model accurately captured the physicochemical property distributions of drug-like small molecules during pretraining. The generated molecules had a low Fréchet ChemNet Distance of 0.728, with a random subset of 10,000 molecules from the training dataset. The lower Fréchet ChemNet Distance score indicates lower similarity between the training set and the molecules from the generative model, thereby suggesting that the model had not memorized the small molecules from the training dataset [34]. Thus all the metrics of our pretrained generative model were in accordance with the desirable thresholds for the benchmark dataset (from the ChEMBL database) [33].

The physicochemical property distributions of the molecules from the pretrained generative model compared with the ChEMBL database are provided in the supplementary data (Supplementary Figure 3).

### Ligand-based drug design using transfer learning

The pretrained generative model discussed above was trained to generate novel drug-like small molecules without any target-specific information. Next, the target information was incorporated into the model through the dataset of target-specific small molecules. The pretrained generative model with knowledge of the SMILES grammar was retrained to capture the features specific to those small molecules that can bind to the target protein of interest (Figure 2B).

In transfer learning (TL), the probability distribution learned from the final few layers is changed in order to bias the generative model toward focusing on a smaller subset of the chemical space. The model was trained with the same set of hyperparameters for 100 epochs in a Tesla K20 GPU. The distribution of the maximum Tanimoto coefficient [35] of the generated small molecules with the training dataset was plotted every ten epochs, to monitor the TL process (Supplementary Figure 4A).

### Property optimization based on predictive model

A dataset of target-specific small molecules and their corresponding property values can be used to train the predictive model. The SMILES strings were preprocessed through the same filters used for the generative model. The deep neural network architecture (Supplementary Figure 2B) of the predictive model consists of bidirectional GRUs as the internal model memory [36]. An embedding layer was used to convert the one-hot-encoded input SMILES strings into a denser representation before passing it through the GRU layers. Two dense layers with rectified linear unit activation were used after the bidirectional GRU layers to extract the property values. Every bidirectional GRU layer was alternated with a dropout layer to avoid overfitting during the model training phase.

The hyperparameters were carefully tuned to achieve a minimum root mean square error in the prediction. The model was trained using mini-batch gradient descent with Adam optimizer [37]. The model was trained up to 500 epochs on a Tesla V100 GPU until convergence.

### Combining the generative & predictive models using reinforcement learning

The generative model obtained after TL was further optimized using reinforcement learning (RL). A regularized policy gradient method was used to train the generative model without catastrophic forgetting of the features learned during pretraining and TL [34]. During RL, the target-specific generative model and the predictive model act as the agent and the critic, respectively (Figure 2B). The agent has learned a prior policy during pretraining, which is the probability distribution over the different symbols at each position of the SMILES string (trajectory/episode). The process of predicting the next symbol in a trajectory given the previous symbols and the model hidden state is the action performed by the agent. Every action of the agent contributes toward an episodic reward calculated at the end of a single sampling iteration. The objective of the policy gradient method is to refine or optimize this prior policy so that the reward obtained is maximum [38].

The reward is a value computed using a reward function defined in terms of the predicted property of the sampled SMILES string from the predictive model. If the predicted property value lies within the desired range, the model is rewarded; otherwise, the model is penalized. The regularization method used here is based on a previous study [27] where two copies of the generative model were considered. The weights of the first copy of the model, termed as the prior, were kept unchanged throughout the joint training phase. The weights of the second copy of the model, termed as the agent, were varied with regularization so that the new policies were highly similar to the prior policy in terms of the learned SMILES grammar, while also ensuring that the generated molecules attained the desirable property. This regularized policy gradient method was used to train the generative model using mini-batch gradient descent with the AMSGrad optimizer [31]. The model was trained for 50 epochs in a Tesla K20 GPU until a visible shift in the property distribution was observed (Supplementary Figure 4B).

### Filtering the generated molecules through physicochemical property filters

The generated small molecules were canonicalized to remove duplicates and molecules similar to the ChEMBL training dataset. The small molecules were further subjected to stringent physicochemical property filters whose thresholds were decided based on a set of known target-specific inhibitors. The various filters applied included synthetic accessibility score ≤5.0, quantitative estimate of drug-likeness >0.4, octanol–water partition coefficient (logP) <6.0, predicted pChEMBL score (bioactivity) >6.0 and molecular weight 400–800 Da. The generated small molecules which passed all the physicochemical property filters were taken for the subsequent steps of filtration and analysis (Figure 2C).

### Application of rule-based filters to remove molecules with undesirable groups

The set of molecules obtained after the application of physicochemical property filters were subjected to screening using the Pan Assay Interference Compounds filter [39], BRENK filter [40], NIH filter [41] and ZINC filter. These filters employ empirically observed rules to avoid toxic and synthetically infeasible subgroups in the small molecules. RDKit was used to apply all four filters on the filtered set of small molecules, and any molecules flagged by at least two of the filters were removed. The remaining compounds were considered for further validation using molecular modeling techniques.

### Validation of the filtered molecules through virtual screening

The final set of molecules was subjected to virtual screening against the 3CL protease of SARS-CoV-2 using AutoDock Vina [25]. The virtual screening score was used to screen the binding affinity of the molecules to the target protein. The molecules with virtual screening score ≤-7.0 were considered as the potential NCEs against the 3CL protease of SARS-CoV-2. The complete pipeline developed to design small molecules specific to any target protein of interest is illustrated in Figure 2.

## Results & discussion

### Generated molecules capture the necessary features from the target-specific small molecule dataset

From the optimized protease-specific generative model obtained after RL, 50,000 small molecules were sampled. The sampled molecules were processed to remove duplicates and ChEMBL-identical molecules, resulting in a dataset of 42,484 molecules. The physicochemical properties of these small molecules were calculated using the RDKit library. It was observed that the properties of the generated molecules were similar to those of the protease-specific small molecule dataset used for TL. However, the generated molecules showed better synthetic accessibility scores (Supplementary Figure 5A) and quantitative estimates of drug-likeness (Supplementary Figure 5B) compared with the TL training dataset.

In order to visualize the subspace of protease-specific small molecules, t-distributed stochastic neighbor embedding (t-SNE) was utilized. Two sets of 1000 molecules each were chosen at random from the molecules generated by the pretrained generative model and model after RL. The input for the t-SNE calculation was 2048-bit extended connectivity fingerprint 4 of the small molecules. These 2000 molecules were used to visualize the chemical space through dimensionality reduction with t-SNE (Supplementary Figure 5C). From the t-SNE plot it can be observed that, although there is some amount of overlap between the chemical spaces of the small molecules from the two models, there is also a distinction between them. The progress of the pretrained generative model into a different subspace of protease-specific molecules is shown along the two dimensions obtained after t-SNE calculations (Supplementary Figure 5C).

To understand the structural features that were captured by the protease-specific generative model during the course of TL and RL, the fragment library in RDKit was used. The average frequencies of various fragments in the molecules generated by the pretrained generative model and the model after RL were computed by sampling 10,000 molecules in ten batches of 1000 molecules each. Because the dataset of viral protease inhibitors consisted of only 2515 molecules, random sampling with replacement was used to augment the dataset for the calculation. The average frequencies of the top ten fragments are shown in Table 1. Interestingly, fragments containing alkyl carbamates, amides, carbonyls and secondary amines had high frequencies among the molecules generated using the protease-specific generative model. It is notable that these groups are commonly found in peptide bonds and in peptidomimetic molecules [42,43], which act as common substrates for proteases; this indicates that RL has effectively learned the features of the protease inhibitors.

**Table 1.** The average frequencies of different fragments commonly present in the ChEMBL dataset, dataset of protease inhibitors and the small molecules sampled from the pretrained generative model and the model after transfer learning and reinforcement learning (protease-specific generative model).

Serial Number	Fragments	ChEMBL dataset	Protease inhibitors dataset	Pretrained generative model	Protease-specific generative model
1	Alkyl carbamates	11.4	408.4	14	305.3
2	C(OH)CCN-Ctert-alkyl or C(OH)CCNcyclic	7.8	135.2	9.9	72.8
3	Aliphatic hydroxyl groups without tert-OH	135.6	711.9	141	708.4
4	Aliphatic hydroxyl groups	159.2	720.3	167.9	744.9
5	Primary amides	28.8	75.7	22.9	71.5
6	Amides	779.8	2328.8	754.2	1799.8
7	Sulfonamides	102.4	309.3	85.5	194.7
8	Secondary amines	891.6	2071.6	831.6	1688.3
9	Carbonyl oxygen without COOH	966.9	2439.8	961.9	1920.9
10	Carbonyl oxygens	1058.8	2542.8	1063.3	2037.5

### Results from the application of physicochemical property-based & rule-based filters to the generated NCEs

The dataset of 42,484 molecules obtained after sampling and removal of redundant and ChEMBL-identical molecules, was subjected to stringent physicochemical property filters (see Methods section). A total of 3960 molecules were found to pass all the five physicochemical property filters. The filtered molecules were further subjected to four empirical rule-based filters to avoid any undesirable subgroups. The percentage of molecules that passed each filter and the most commonly observed substructure flags are tabulated in the supplementary data (Supplementary Table 1). It was observed that most of the molecules were flagged in a similar way by both the NIH and the BRENK filters, due to overlap of the rules defined by each database.

As mentioned above, molecules that were flagged by at least two of the four filters were removed from further analysis, resulting in a dataset of 3651 molecules from 3960. These molecules were further screened for their affinity toward the binding site of the 3CL protease of SARS-CoV-2. The resulting dataset of 1267 molecules with virtual screening scores below -7.0 was considered for further analysis (Supplementary Data 2).

### Generated NCEs have similarity to protease inhibitors currently in clinical trials & show better virtual screening scores

**Figure 3.**
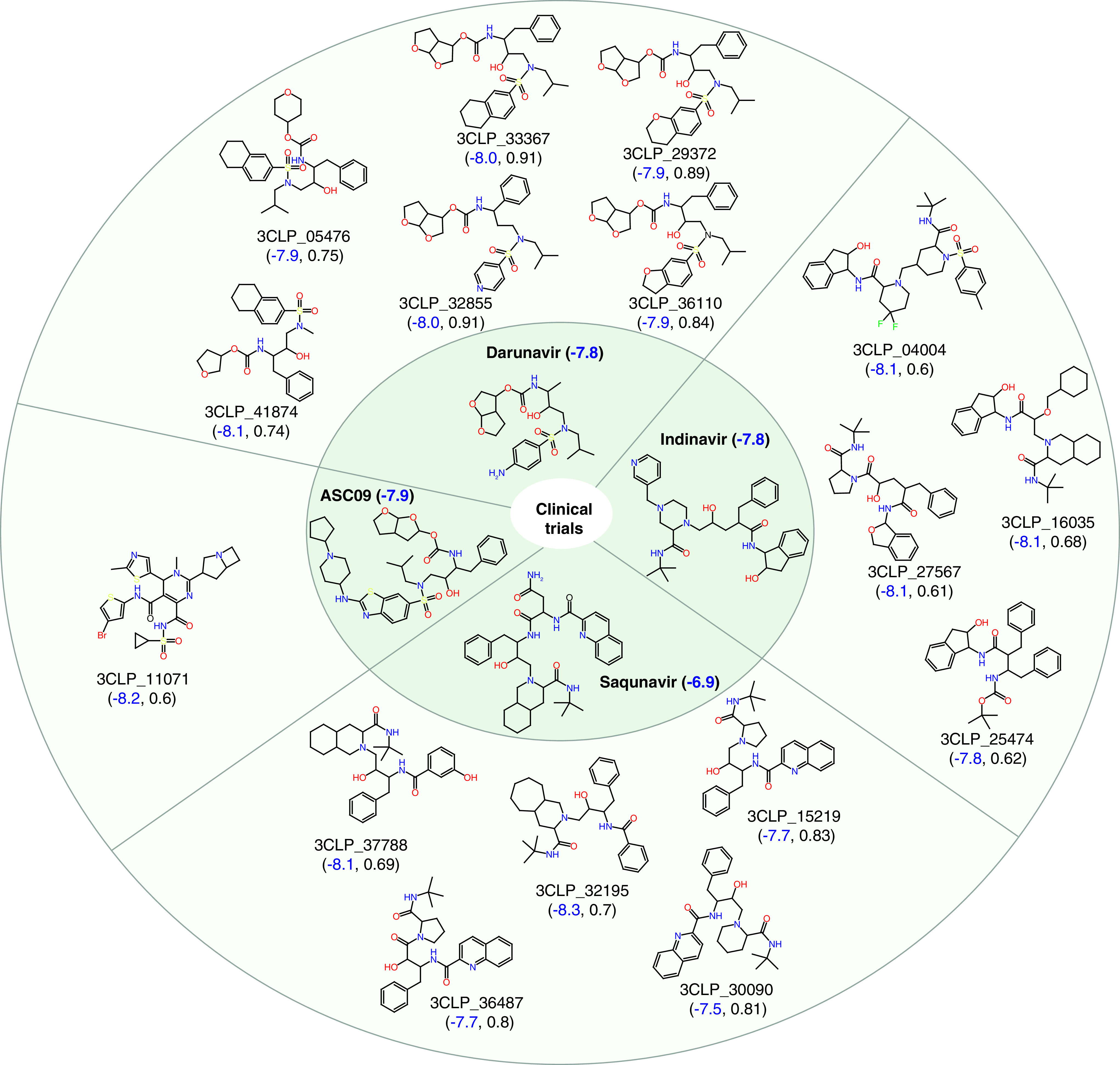
New chemical entities similar to the HIV protease inhibitors. Structurally similar new chemical entities (NCEs) with better virtual screening score than existing HIV protease inhibitors (ASC09, darunavir, indinavir and saquinavir) that are in clinical trials against SARS-CoV-2. The virtual screening score and the Tanimoto similarity of the NCEs are shown in blue and black within the bracket. The virtual screening scores of existing HIV protease inhibitors in clinical trials are also shown in blue.

AutoDock Vina [25] was used to calculate the virtual screening scores of existing HIV protease inhibitors (ASC09, darunavir, indinavir, lopinavir, ritonavir and saquinavir) in clinical trials against SARS-CoV-2 [14]. The NCEs generated by the model with good similarity to the HIV protease inhibitors, and with similar or higher virtual screening scores, are shown in Figure 3. The virtual screening scores were observed to be in the range of -8.3 to -7.5. The generated NCE 3CLP_32855 was found to have the highest Tanimoto similarity (0.91) [35] with darunavir. Several of the molecules shown in Figure 3 had high similarity to darunavir, indinavir and saquinavir.

### Generated NCEs with high virtual screening scores

**Figure 4.**
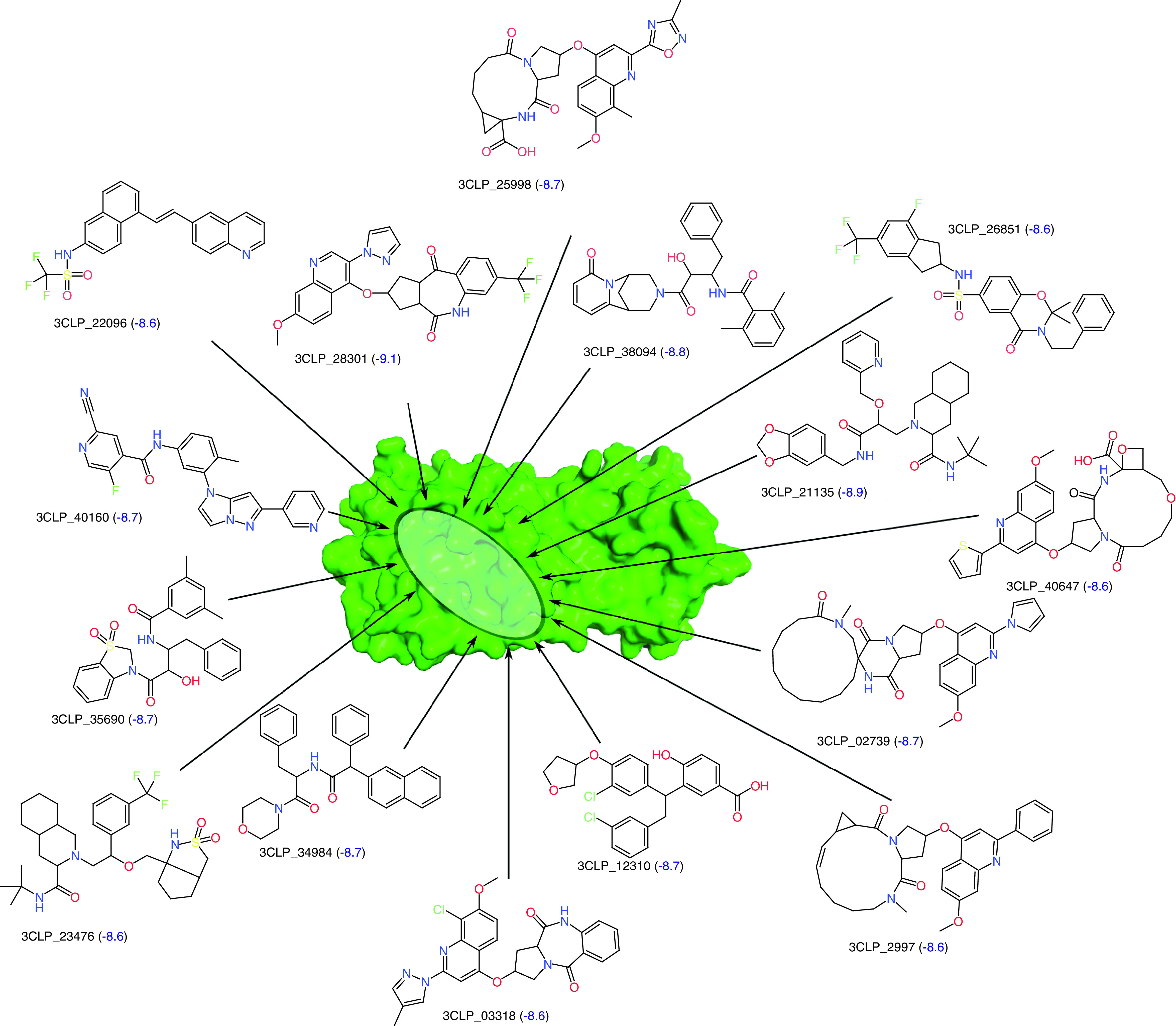
New chemical entities with highest virtual screening score. Generated new chemical entities with highest virtual screening score (shown in blue) against the 3CL protease of SARS-CoV-2. The 3CL protease is shown in surface representation. The active site of the 3CL protease is highlighted using an ellipse.

The first set of potential NCEs for synthesis and testing against SARS-CoV-2 were shortlisted using a virtual screening score cutoff of -8.5 (Figure 4). The generated NCE 3CLP_28301 was found to have the highest virtual screening score of -9.1. Five of the top 15 compounds were found to have both a high virtual screening score and higher similarity to the existing protease inhibitors (tanimoto coefficient (TC) >0.80) collected from the ChEMBL database. The generated molecules were found to be structurally diverse, as indicated by their internal diversity [44] of 0.61. The various physicochemical properties of the 31 small molecules (shown in Figures 3 & 4) are available in the supplementary data 1 (Supplementary Table 2). The best docking poses for the top four compounds from Figure 4 are shown in Supplementary Figure 6.

### Comparison of generated molecules with natural products

**Figure 5.**
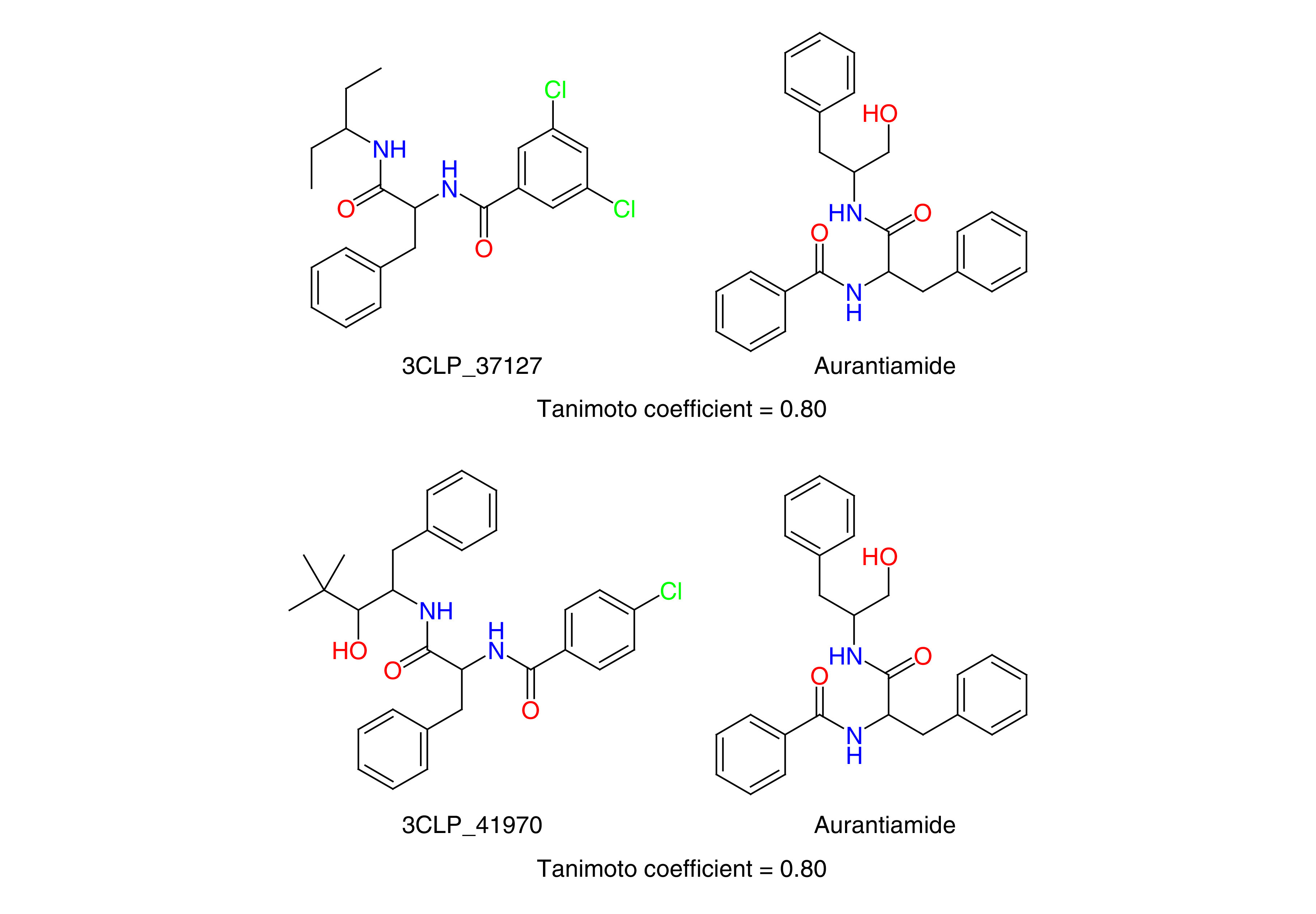
Comparison of generated new chemical entities and natural products. 2D structures of generated new chemical entities (NCEs, left) and Aurantiamide (right). The similarity between the NCEs and Aurantiamide was quantified using the Tanimoto coefficient.

The NCEs generated by the generative model were compared with the SymMap database of traditional medicines [45]. Based on this comparison, two of the NCEs were found to be similar to the natural product aurantiamide (Figure 5). However, the virtual screening scores of these molecules were marginally lower compared with the molecules reported in Figures 3 & 4. Aurantiamide is a phytochemical extracted from the herb *Baphicacanthus cusia*, which is widely used for the treatment of cold, fever and influenza [46] and is also referred to as Nees, Bremek or *Strobilanthes cusia*. This herb is broadly found in the southern districts of China, Bangladesh, India and Myanmar [47]. In India, aurantiamide is also extracted from *Piper aurantiacum* [48].

The different pharmacokinetic and toxicity properties of the 33 potential NCEs identified by this study, and of 6 HIV protease inhibitors (ASC09, darunavir, indinavir, lopinavir, ritonavir and saquinavir) were computed using SwissADME [49], ToxTree (v3.1.0) [50] and pkCSM [51]. It was observed that most of the NCEs have good bioavailability and do not permeate through the blood–brain barrier. None of the NCEs showed genotoxicity as predicted by either ToxTree (v3.1.0) [50] or pkCSM [51] (see Supplementary Data 1, section 1). Based on the above results, it can be inferred that the designed molecules can be considered for synthesis and testing against the 3CL protease of SARS-CoV-2.

## Conclusion

In this work we have generated potential NCEs to inhibit the 3CL protease of SARS-CoV-2. By leveraging the power of recurrent neural networks, the inherent grammar of small molecules was captured to design novel small molecules. TL, followed by RL, helped to design protease-specific small molecules with optimized properties. The application of several stringent physicochemical property filters and the removal of potentially toxic and undesirable subgroups helped to refine the generated set of small molecules. The filtered set of small molecules were further screened at the active site of 3CL protease to identify a ranked list of potential drug-like NCEs. The results show that the generative model can generate novel NCEs with high similarity to existing HIV protease inhibitors, while also being able to bind better to the 3CL protease (Figures 3 & 4). Two of the designed NCEs were also observed to have high similarity to the phytochemical aurantiamide, which has known antiviral properties. The complete set of promising small molecules has been provided in the Supplementary Data 2 to facilitate testing against SARS-CoV-2.

## Future perspective

Drug discovery is a long, expensive and complex process with a very low success rate. Recent advances in the field of AI have fueled the emergence of new methods to accelerate the drug discovery process. The vast chemical space that is available to sample drug-like molecules can be efficiently explored using a combination of AI-based methods. In this work, we propose 33 NCEs that can be tested against the 3CL protease of SARS-CoV-2. The generalized method proposed in this work can significantly accelerate the drug discovery process.

**Summary points**

- Deep learning-based generative and predictive models were developed for *de novo* design of small molecules.
- The proposed method was used to design new chemical entities for the 3CL protease of SARS-CoV-2.
- Transfer learning helped the generative model to learn the features of the known protease-specific inhibitors.
- Reinforcement learning was used to optimize the property of the new chemical entities (NCEs).
- A total of 31 potential NCEs were identified. Some of these NCEs were similar to the HIV protease inhibitors with better binding affinity toward 3CL protease.
- Two potential NCEs similar to a natural product, aurantiamide, were identified.
