# Supplementary material for: De novo design of new chemical entities for SARS-CoV-2 using artificial intelligence
Source: Future Med Chem. 2021 Feb 16:10.4155/fmc-2020-0262. doi: 10.4155/fmc-2020-0262 (PMC7888348; doi:10.4155/fmc-2020-0262)
Supplement: Supplementary file 1 [file Supplementary_data_1.doc]

**Supplementary data 1**

**Table S1** Percentage of molecules which passed each of the rule-based filters and the common errors flagged by each filter for the input set of molecules from the generative model.

| **Rule-based filter** | **Qualified molecules (%)** | **Common structural flags (Rule notations as defined in RDKit)** |
| --- | --- | --- |
| PAINS | 98.48 | anil_no_alk, anil_di_alk, catechol, mannich, imine_one |
| BRENK | 78.83 | 2-halo_pyridine, aldehyde, Michael_acceptor_1, Aliphatic_long_chain, alkyl_halide, aniline, isolated_alkene, beta-keto/anhydride, catechol, charged_oxygen_or_sulfur_atoms, cumarine, cyanamide, diketo_group, heavy_metal, het-C-het_not_in_ring, hydantoine, imine_1, imine_2, Oxygen-nitrogen_single_bond, iodine, isolated_alkene, phthalimide, triple_bond, stilbene, Sulfonic_acid_1, Sulfonic_acid_2, sulphur_nitrogen_single_bond, thioester, thiol_2, Three-membered_heterocycle |
| NIH | 93.23 | 2halo_pyridine_3EWG, aldehyde, alpha_dicarbonyl, betalactam, boron_warhead, crown_ether, cyanohydrin, keto_def_heterocycle, non_ring_acetal, non_ring_ketal, secondary_halide_sulfate, sulf_D2_nitrogen, thio_hydroxamate |
| ZINC | 96.59 | Fluorines, Non-Hydrogen_atoms |

**Table S2** Physico-chemical properties of the top 31 NCEs shortlisted for synthesis and testing (NCEs from Fig. 3 and Fig. 4).

| **Molecule ID** | **logP*** | **MW***  **(Da)** | **HBA*** | **HBD*** | **NRB*** | **TPSA***  **(Å^2^)** | **Benzene count** | **QED*** | **SAS*** |
| --- | --- | --- | --- | --- | --- | --- | --- | --- | --- |
| 3CLP_28301 | 5.06 | 522.2 | 7 | 1 | 4 | 95.3 | 4 | 0.40 | 3.90 |
| 3CLP_21135 | 3.81 | 550.3 | 7 | 2 | 9 | 102.0 | 2 | 0.49 | 3.99 |
| 3CLP_38094 | 2.74 | 485.2 | 5 | 2 | 6 | 91.6 | 3 | 0.56 | 4.41 |
| 3CLP_02739 | 4.23 | 587.3 | 7 | 1 | 4 | 106.0 | 3 | 0.49 | 4.94 |
| 3CLP_25998 | 2.40 | 535.2 | 9 | 2 | 5 | 157.0 | 3 | 0.50 | 4.64 |
| 3CLP_34984 | 4.56 | 478.2 | 3 | 1 | 7 | 58.6 | 4 | 0.42 | 2.90 |
| 3CLP_35690 | 2.78 | 478.2 | 5 | 2 | 6 | 103.8 | 3 | 0.57 | 3.35 |
| 3CLP_12310 | 5.94 | 472.1 | 4 | 2 | 7 | 76.0 | 3 | 0.44 | 3.22 |
| 3CLP_29997 | 5.09 | 525.3 | 5 | 0 | 4 | 72.0 | 3 | 0.44 | 4.35 |
| 3CLP_22096 | 5.82 | 428.1 | 3 | 1 | 4 | 59.1 | 4 | 0.42 | 2.46 |
| 3CLP_03318 | 4.01 | 503.1 | 7 | 1 | 4 | 98.6 | 4 | 0.45 | 3.64 |
| 3CLP_40647 | 2.67 | 567.2 | 9 | 2 | 5 | 136.5 | 3 | 0.48 | 4.79 |
| 3CLP_26851 | 5.10 | 562.2 | 4 | 1 | 6 | 75.7 | 3 | 0.42 | 3.40 |
| 3CLP_23476 | 5.03 | 599.3 | 5 | 2 | 7 | 87.7 | 1 | 0.46 | 4.84 |
| 3CLP_40160 | 4.15 | 437.1 | 7 | 1 | 4 | 100.9 | 5 | 0.46 | 2.97 |
| 3CLP_32195 | 4.57 | 519.3 | 4 | 3 | 8 | 81.7 | 2 | 0.48 | 3.68 |
| 3CLP_11071 | 3.26 | 638.0 | 10 | 2 | 7 | 124.1 | 2 | 0.48 | 4.72 |
| 3CLP_41874 | 2.67 | 502.2 | 6 | 2 | 9 | 105.2 | 2 | 0.55 | 3.52 |
| 3CLP_16035 | 4.52 | 553.4 | 5 | 3 | 8 | 90.9 | 1 | 0.44 | 4.31 |
| 3CLP_27567 | 2.85 | 507.3 | 5 | 3 | 8 | 108.0 | 2 | 0.51 | 3.92 |
| 3CLP_04004 | 3.55 | 646.3 | 6 | 3 | 7 | 119.1 | 2 | 0.42 | 4.33 |
| 3CLP_37788 | 3.89 | 521.3 | 5 | 4 | 8 | 101.9 | 2 | 0.42 | 3.79 |
| 3CLP_32855 | 2.19 | 533.2 | 8 | 2 | 11 | 127.3 | 2 | 0.45 | 4.02 |
| 3CLP_29372 | 3.67 | 586.3 | 7 | 2 | 11 | 114.4 | 2 | 0.41 | 4.07 |
| 3CLP_33357 | 3.12 | 588.3 | 8 | 2 | 11 | 123.6 | 2 | 0.41 | 4.15 |
| 3CLP_05476 | 4.09 | 558.3 | 6 | 2 | 11 | 105.2 | 2 | 0.43 | 3.41 |
| 3CLP_36110 | 2.73 | 574.2 | 8 | 2 | 11 | 123.6 | 2 | 0.42 | 4.13 |
| 3CLP_25474 | 4.76 | 500.3 | 4 | 3 | 8 | 87.7 | 3 | 0.42 | 3.51 |
| 3CLP_36487 | 2.84 | 502.3 | 5 | 3 | 7 | 111.6 | 3 | 0.46 | 3.33 |
| 3CLP_15219 | 3.71 | 502.3 | 5 | 3 | 8 | 94.6 | 3 | 0.44 | 3.37 |
| 3CLP_30090 | 3.32 | 488.3 | 5 | 3 | 8 | 94.6 | 3 | 0.45 | 3.36 |

*logP – Partition coefficient; MW – Molecular Weight; HBA – Hydrogen Bond Acceptor; HBD – Hydrogen Bond Donor; TPSA – Topological Polar Surface Area; QED – Quantitative Estimate of Drug-likeness; SAS – Synthetic Accessibility Score.

Section 1. **Pharmacokinetics and Toxicity of the top 33 molecules and the HIV protease inhibitors**

The critical pharmacokinetic and toxicity properties of the top 33 potential NCEs and the known HIV protease inhibitors (ASC09, Darunavir, Indinavir, Lopinavir, Ritonavir, and Saquinavir) were compared. The different pharmacokinetic properties and toxicity was predicted using SwissADME [1], pkCSM [2] and ToxTree (v3.1.0) [3]. The results from the web servers are provided in the supplementary material 2.

a) **Bioavailability**: 90% of the 33 molecules had a bioavailability score of 0.55 from SwissADME indicating sufficient rat bioavailability. Surprisingly, only 50% of the 6 HIV protease inhibitors had sufficient rat bioavailability (supplementary material 2).

b) **Blood brain barrier (BBB) permeability**: 90% of the 33 molecules were predicted by SwissADME to not cross the BBB, while 94% of the molecules were predicted by pkCSM to not cross the BBB. All 6 HIV protease inhibitors were also predicted to not cross the BBB by both SwissADME and pkCSM.

c) **Toxicity**: The genotoxic carcinogenicity (Ames test) was predicted using ToxTree [3] and pkCSM [2]. It was observed that, out of the top 33 NCEs, 76% and 89% molecules do not cause genotoxic carcinogenicity as predicted by ToxTree and pkCSM, respectively (supplementary material 2). However, all molecules passed the genotoxicity test by either one of the methods. In comparison, according to ToxTree, 50% of the HIV protease inhibitors do not cause genotoxic carcinogenicity while pkCSM predicted that none of the HIV protease inhibitors cause genotoxic carcinogenicity.

Although most of the molecules pass the toxicity test with desired pharmacokinetic properties by atleast one of the methods, these results indicate that the results from different pharmacokinetics and toxicity prediction tools differ widely, and more demanding computational tools or experimental methods are required to determine the properties of these molecules.

**Supplementary figures**


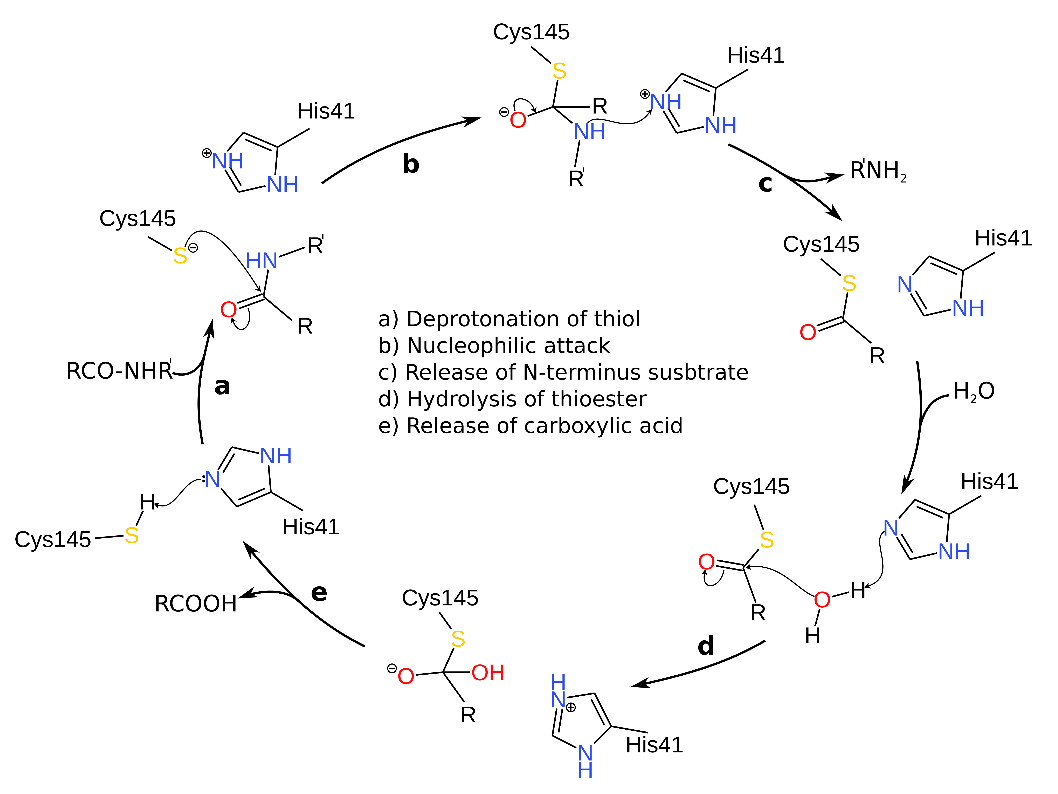
**Fig. S1** The proteolysis mechanism of 3CL protease in SARS-CoV-2 [4,5]. His41, Cys145 and a water molecule in the active site plays an important role in the catalysis.


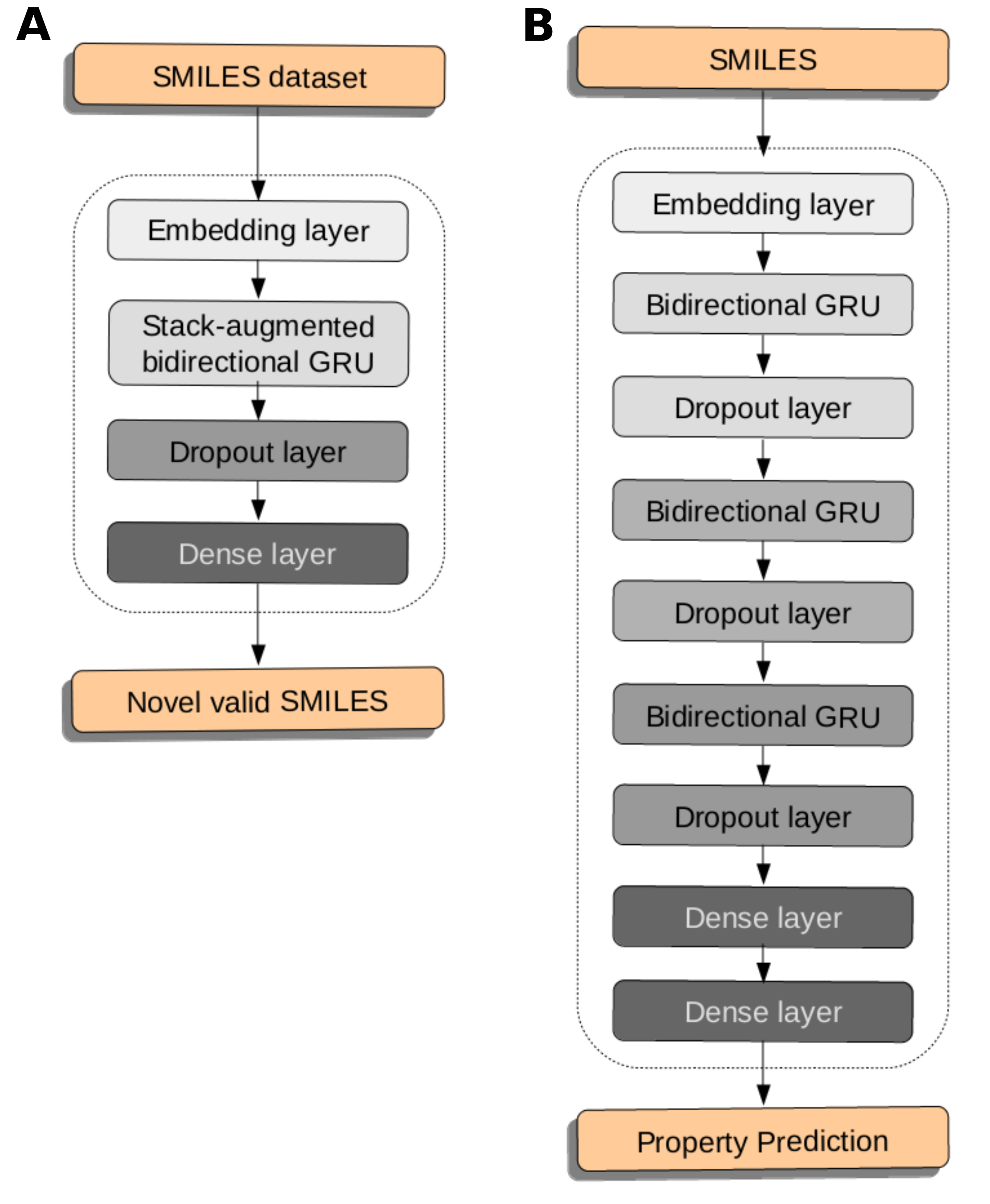


**Fig. S2** The deep neural network architecture of the (**A**) Generative model and (**B**) Predictive model.


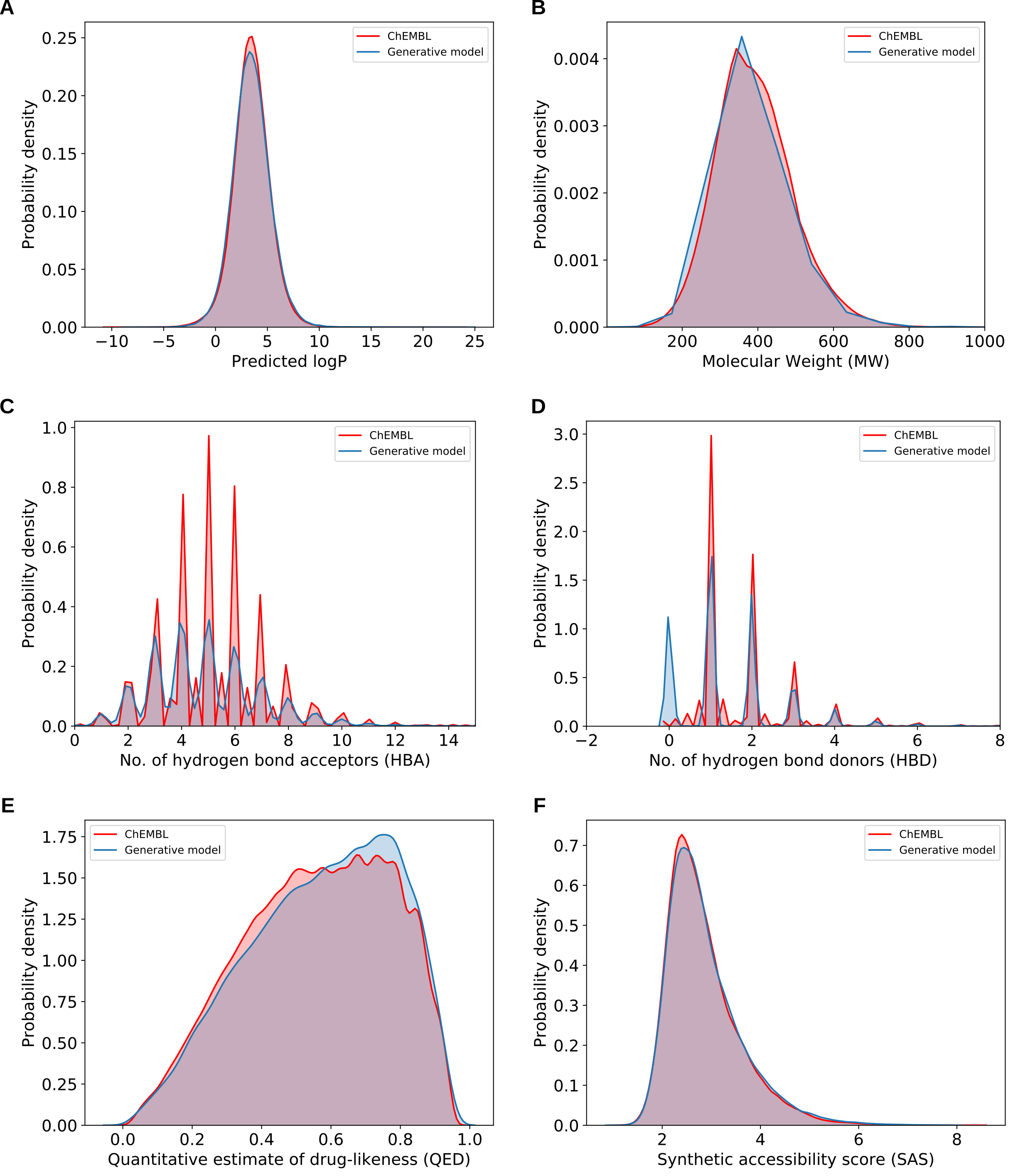
**Fig. S3** The physico-chemical property distributions of the pre-trained generative model compared with the distributions for the training dataset from the ChEMBL database. (**A**) Octanol-water partition coefficient (logP), (**B**) Molecular weight (MW), (**C**) Number of hydrogen bond acceptors (HBA), (**D**) Number of hydrogen bond donors (HBD), (**E**) Quantitative estimate of drug-likeness (QED) and (**F**) Synthetic accessibility score (SAS).


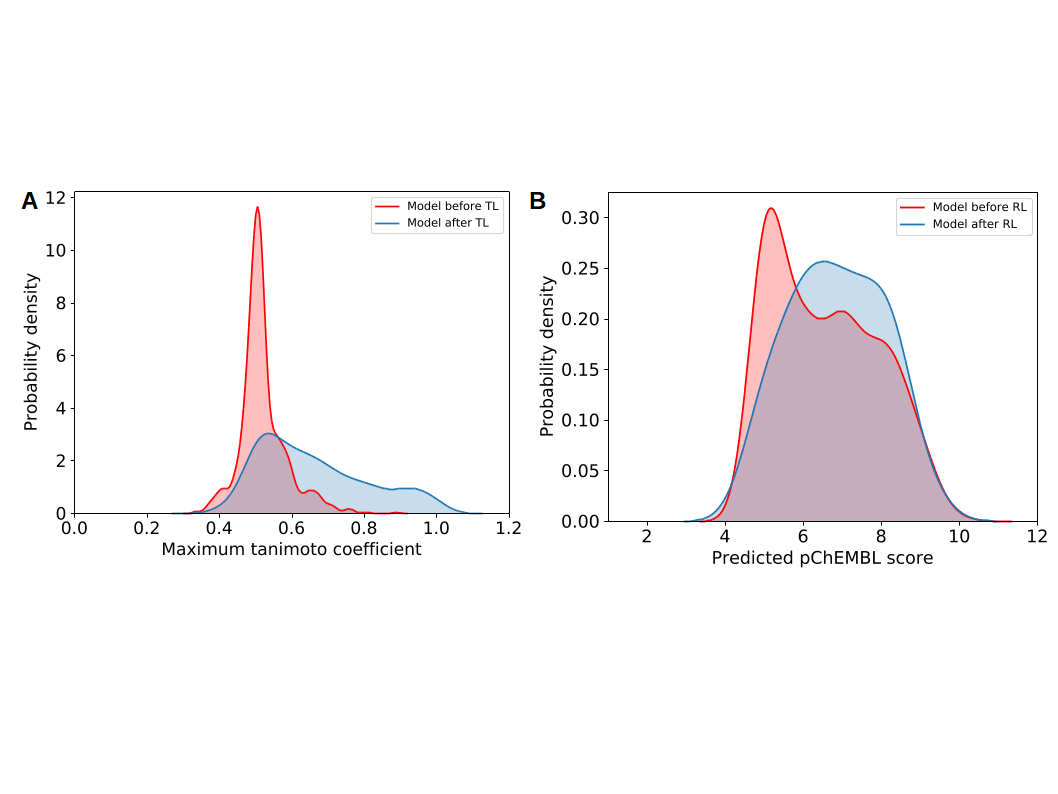
**Fig. S4** (**A**) The distribution of the maximum Tanimoto coefficient of the molecules from the generative model with the protease-specific transfer learning training dataset after 100 epochs. (**B**) The distribution of the predicted pChEMBL scores of the molecules from the protease-specific generative model after 50 epochs of reinforcement learning.


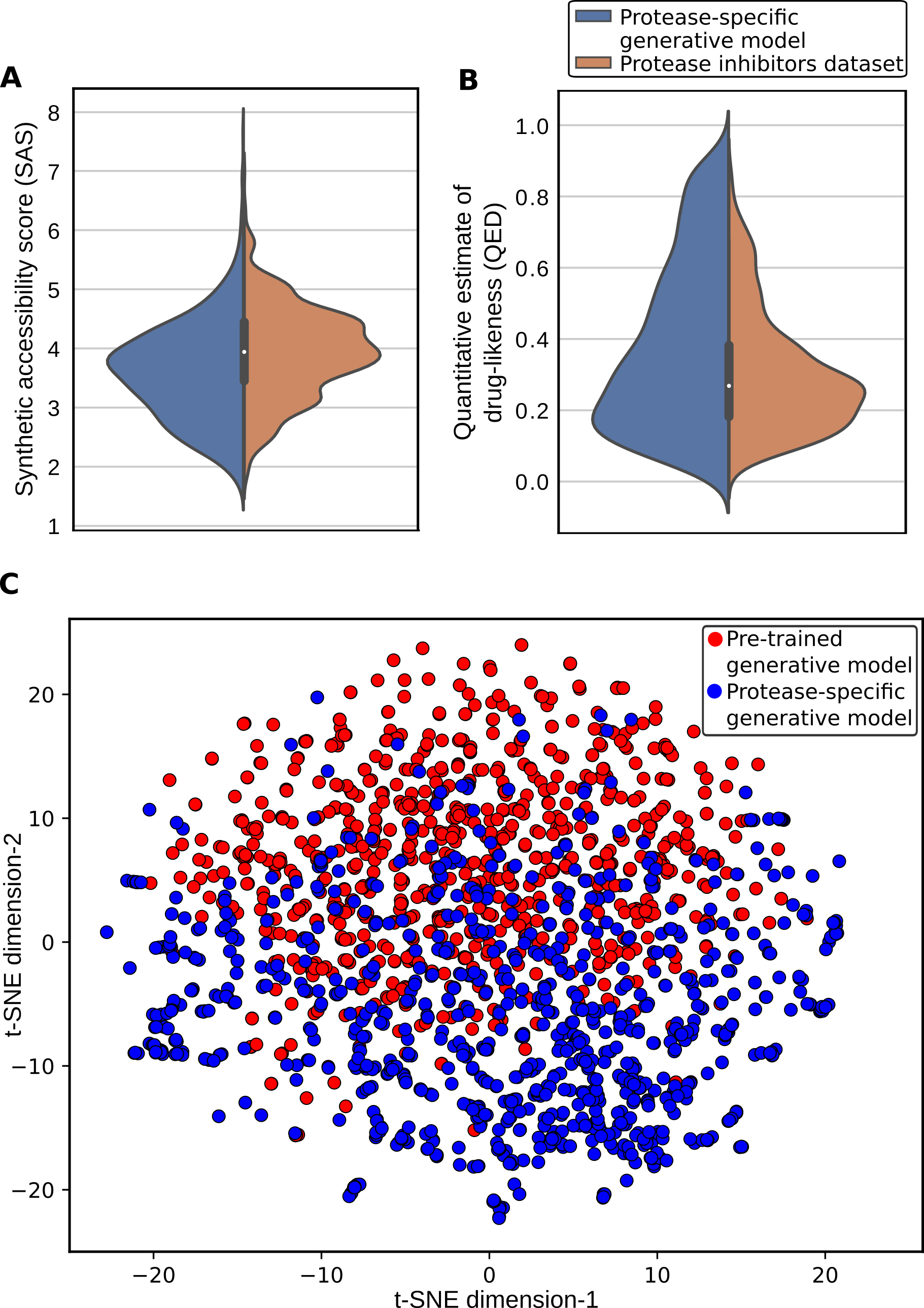
**Fig. S5** Violin plot of the distribution of (**A**) SAS and (**B**) QED values of the small molecules from the protease-specific generative model (shown in blue) and the protease inhibitors dataset (shown in brown). A significant fraction of the generated molecules have optimal QED (>0.5) and SAS (<4) values compared to the protease inhibitors dataset. (**C**) Visualization of the chemical space explored by the generative model using t-SNE. The red points indicate molecules sampled from the pre-trained generative model and blue points indicate molecules generated by the protease-specific generative model obtained after RL.


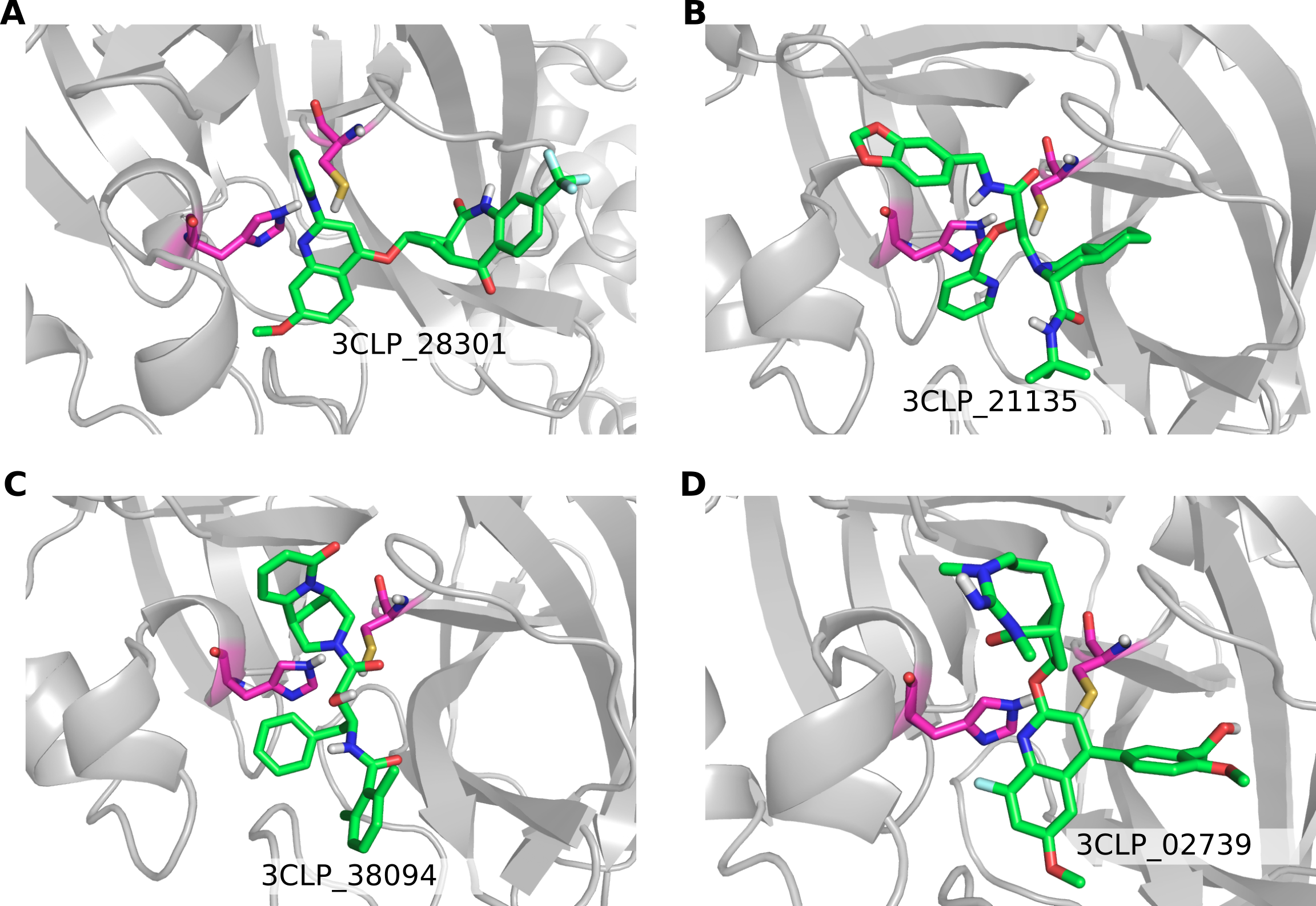


**Fig. S6** The best docking pose of the top 4 compounds generated using AutoDock Vina (**A-D**). The SARS-CoV-2 3CL protease is shown in cartoon representation while the top four compounds are shown as green sticks. The active site residues, His41 and Cys145, are shown as magenta sticks.

**References**

(1) Daina A, Michielin O, Zoete V. SwissADME: a free web tool to evaluate pharmacokinetics, drug-likeness and medicinal chemistry friendliness of small molecules. *Sci. Rep*. 7, 42717 (2017).

(2) Pires DE, Blundell TL, Ascher DB. pkCSM: Predicting Small-Molecule Pharmacokinetic and Toxicity Properties Using Graph-Based Signatures. *J. Med. Chem.* 58 (9), 4066-4072 (2015).

(3) Patlewicz G, Jeliazkova N, Safford RJ, Worth AP, Aleksiev B. An evaluation of the implementation of the Cramer classification scheme in the Toxtree software. *SAR QSAR Environ. Res.* 19(5-6), 295-524 (2008).

(4) Pillaiyar T, Manickam M, Namasivayam V, Hayashi Y, Jung SH. An overview of severe acute respiratory syndrome–coronavirus (SARS-CoV) 3CL protease inhibitors: Peptidomimetics and small molecule chemotherapy. *J. Med. Chem.* 59 (14), 6595-6628 (2016).

(5) Muramatsu T, Takemoto C, Kim YT et al. SARS-CoV 3CL protease cleaves its C terminal autoprocessing site by novel subsite cooperativity. *Proc. Natl. Acad. Sci.* 113 (46), 12997-13002 (2016).
